# Ambient and controlled exposures to particulate air pollution and acute changes in heart rate variability and repolarization

**DOI:** 10.1038/s41598-019-38531-9

**Published:** 2019-02-13

**Authors:** Susanne Breitner, Annette Peters, Wojciech Zareba, Regina Hampel, David Oakes, Jelani Wiltshire, Mark W. Frampton, Philip K. Hopke, Josef Cyrys, Mark J. Utell, Cathleen Kane, Alexandra Schneider, David Q. Rich

**Affiliations:** 1 Helmholtz Zentrum München–German Research Center for Environmental Health, Institute of Epidemiology, Neuherberg, Germany; 2 Ludwig-Maximilians-Universität München, IBE-Chair of Epidemiology, Munich, Germany; 3 University of Rochester Medical Center, Rochester, New York USA; 4 Clarkson University, Potsdam, New York USA

## Abstract

Previous studies have reported increased risks of myocardial infarction in association with elevated ambient particulate matter (PM) in the previous hour(s). However, whether PM can trigger mechanisms that act on this time scale is still unclear. We hypothesized that increases in PM are associated with rapid changes in measures of heart rate variability and repolarization. We used data from panel studies in Augsburg, Germany, and Rochester, New York, USA, and two controlled human exposure studies in Rochester. Data included ECG recordings from all four studies, controlled exposures to (concentrated) ultrafine particles (UFP; particles with an aerodynamic diameter <100 nm) and ambient concentrations of UFP and fine PM (PM_2.5_, aerodynamic diameter <2.5 μm). Factor analysis identified three representative ECG parameters: standard deviation of NN-intervals (SDNN), root mean square of successive differences (RMSSD), and T-wave complexity. Associations between air pollutants and ECG parameters in the concurrent and previous six hours were estimated using additive mixed models adjusting for long- and short-term time trends, meteorology, and study visit number. We found decreases in SDNN in relation to increased exposures to UFP in the previous five hours in both of the panel studies (e.g. Augsburg study, lag 3 hours: −2.26%, 95% confidence interval [CI]: −3.98% to −0.53%; Rochester panel study, lag 1 hour: −2.69%; 95% CI: −5.13% to −0.26%) and one of the two controlled human exposure studies (1-hour lag: −13.22%; 95% CI: −24.11% to −2.33%). Similarly, we observed consistent decreases in SDNN and RMSSD in association with elevated PM_2.5_ concentrations in the preceding six hours in both panel studies. We did not find consistent associations between particle metrics and T-wave complexity. This study provided consistent evidence that recent exposures to UFP and PM_2.5_ can induce acute pathophysiological responses.

## Introduction

Multiple studies have reported that short-term elevations in ambient particulate matter (PM) might trigger acute coronary events including myocardial infarctions (MI)^1–4^, with some even suggesting that ambient PM might trigger MI within one or two hours^1,5^. However, other studies have not reported such rapid associations^6,7^. We and others have also reported associations between exposure to traffic in the previous hour and an increased risk of MI^8^ and episodes of ventricular arrhythmia^9,10^.

Pathways thought to be important in the association between ambient PM and acute cardiovascular events include systemic inflammation, autonomic dysfunction, endothelial dysfunction, and local inflammation and oxidative stress^11,12^. Decreased heart rate variability (HRV) has been associated with cardiac morbidity and mortality and is often used as a marker of autonomic dysfunction in the assessment of air pollution impacts on cardiac autonomic control^13^. Abnormalities in T-wave morphology and repolarization reflect changes in the myocardial substrate; they have been found to also precede adverse cardiovascular events and increase the risk for coronary deaths^14,15^.

Only a small number of studies have investigated whether exposure to particulate air pollution is associated with HRV responses during the subsequent hours^16–19^. For example, among patients with coronary artery disease, decreases in HRV parameters were associated with exposures to PM_2.5_ (PM with an aerodynamic diameter <2.5 µm) or black carbon (BC) in the previous two hours^16^. HRV indices were also associated with individual-level PM_2.5_ exposures on a one- to six-hour basis^17^. Further, a number of studies of car commuters, cyclists, taxi-drivers or walkers have reported (small) acute HRV changes of micro-environmental (e.g. cycling on high- and low traffic routes, walking along roadsides, inside vehicles) exposures to traffic-related air pollution^20–24^. Evidence on the relationship between elevated levels of PM and T-wave complexity is scarce^25,26^. Henneberger, *et al*.^25^ detected an increase in T-wave complexity in association with increases in the 6-hour averages of PM_2.5_ in stable ischemic heart disease patients; a controlled human exposure study found trends to increased variability of T-wave complexity after exposure to elemental carbon ultrafine particles (UFP; particles with a diameter <100 nm)^26^. So far, however, it remains unclear whether there is consistency in the health effects of exposures to ambient and controlled PM concentrations.

Last, studies have also identified subgroups (e.g. patients with previous myocardial infarction or diabetes, individuals with genetic susceptibility to oxidative stress) which may be more susceptible to the harmful effects of particulate air pollution than the general population^11,12^.

We used data from four completed studies: (1) a panel study of patients with type 2 diabetes, participants with impaired glucose tolerance, or healthy individuals with a potential genetic pre-disposition which could affect detoxifying and inflammatory pathways conducted in Augsburg, Germany, (2) a panel study of patients with a recent coronary event (MI or unstable angina) participating in a cardiac rehabilitation program in Rochester, New York, (3) a controlled human exposure study of patients with diabetes in Rochester, New York, and (4) a controlled human exposure study of healthy adults in Rochester, New York. We selected the four studies as all of them had data on UFP and ECG measurements available. Although there exists a large number of studies on the associations between PM_2.5_ and HRV, research on the effects of hourly UFP exposure on HRV or other ECG markers is scarce. We further selected the two epidemiological panel studies as they provided the opportunity to investigate real life-mixtures of ambient air pollution with a special focus on ultrafine and fine PM and their cardiac impact in selected populations. Using the two controlled human exposure studies, we aimed to validate the findings of the epidemiologic panel studies under ideal conditions. Using a discovery/replication approach^27^ to make inference across analyses conducted in all 4 studies, we hypothesized that increased hourly PM concentrations would be associated with decreased SDNN, RMSSD, and increased T-wave complexity.

## Methods

### Study population

Our study population included two panel studies (Augsburg panel and Rochester REHAB studies) and two controlled human exposure studies (Rochester UPCON and UPDIABETES studies). All four study populations are briefly described below; for more detailed descriptions, see Hampel, *et al*.^28^, Rich, *et al*.^29^, Rich, *et al*.^27^, and Stewart, *et al*.^30^.

This study was approved by the Ethics Commission of the Bavarian Chamber of Physicians (Bayerische Landesärztekammer) and the Research Subjects Review Board at the University of Rochester Medical Center in Rochester, New York; all participants signed informed consent prior to enrollment. All methods were carried out in accordance with relevant guidelines and regulations.

#### Augsburg Panel Study

The protocol for the Augsburg panel study has been described in detail elsewhere^28^. Briefly, we enrolled 64 participants having either a diagnosed type 2 diabetes (T2D; n = 32) or impaired glucose tolerance (IGT; n = 32), and 46 healthy individuals with a potential genetic pre-disposition which could affect detoxifying and inflammatory pathways between March 2007 and December 2008. For each participant, information on life-style, additional diseases and medication intake were collected at a baseline visit. All subjects participated in up to four ECG recordings; visits were scheduled every four to six weeks on the same weekday and at the same time of the day. Participants carrying a 12-lead Mortara H12 digital Holter recorder (Mortara Instruments, Milwaukee, WI) left the study center to pursue their daily routines and returned after approximately six hours. In total, 364 ECG recordings of approximately five to six hours were available for the analyses.

#### Rochester REHAB Study

The Rochester REHAB study protocol has been described elsewhere^29,31,32^. Briefly, REHAB comprised 76 patients with a recent coronary event (MI or unstable angina) who participated in a standard cardiac rehabilitation program at the University of Rochester from June 2006 to November 2009. Subjects participated in up to 20 rehabilitation sessions scheduled twice a week over a 10-week period; each session also included 30 to 45 minutes of exercise. During each session, participants underwent 3-lead (modified V2, V5, and AVF) Holter ECG recordings (Vision Premier, Burdick, Milwaukee, WI) of approximately two to three hours, including a 10-minute resting ECG before the exercise period. Resting recordings were obtained for baseline pre-exercise information. Three subjects had to be excluded for the hourly analysis because of inadequate recordings, leaving 73 participants with ECG recordings.

#### Rochester UPCON and UPDIABETES Studies

Study populations and protocols for both studies have been described previously^27,30,33^. Briefly, both studies were double-blind, randomized crossover studies of 20 healthy lifetime nonsmokers (UPCON Study) and 19 individuals having type 2 diabetes as defined by the World Health Organization (UPDIABETES Study). Participants of both studies were admitted to the Clinical Research Center at the University of Rochester the day before exposure and stayed overnight, to reduce confounding effects of ambient air pollution exposures. The UPCON participants were exposed twice while at rest for two hours: either to filtered air or to outdoor ambient UFP that were concentrated using a Harvard UFP concentrator system. UPDIABETES subjects inhaled either freshly generated elemental carbon UFP or filtered air, also for two hours at rest. ECG recordings in both studies started two hours before the exposure, for a total of 24 hours in the UPCON and for 48 hours in the UPDIABETES study. One UPCON and one UPDIABETES participant had to be excluded for the 1-hour analyses described below due to missing ECG recordings.

### Exposure assessment

#### Augsburg Panel Study

Among others, ambient concentrations of UFP, accumulation mode particles (AMP; aerodynamic diameter in the size range 100 to 500 nm), PM_2.5_, and black carbon (BC) were monitored at an urban background site located on the campus of the University of Applied Sciences Augsburg in Augsburg, Germany. Data on meteorological variables including air temperature, relative humidity, and barometric pressure were collected at the same site. All of the particle metrics or meteorological data were available on an hourly basis.

#### Rochester REHAB Study

Ambient hourly concentrations of UFP and AMP were measured at the Cardiac Rehabilitation Center between June 2006 and November 2009. In addition, hourly PM_2.5_ mass and BC concentrations, and meteorological variables including air temperature and relative humidity were monitored at the New York State Department of Environmental Conservation site in Rochester, New York throughout the study period.

#### UPCON Study

Exposures to either concentrated ambient UFP or filtered air were conducted in a specially designed exposure chamber between November 2006 and June 2008. The chamber - made of plexiglass and stainless steel - was maintained at 12 cm H_2_O relative to atmospheric pressure. Exposures were generated using a Harvard Ultrafine Concentrated Ambient Particle System (HUCAPS). For filtered-air exposures, a HEPA filter was used at the HUCAPS outlet. Participants received approximately 50–60 L/min of HUCAPS output air via a venturi-type face mask (Hudson RCI, Teleflex Medical, Research Triangle Park, NC) covering the nose and mouth. During exposure, particle number concentration (PNC; used as a surrogate for UFP) was measured outdoors at the HUCAPS intake (approximately 20 meters from the roadway) as well as at the face mask. Final exposures to concentrated ambient UFP varied depending on the ambient particle concentrations. Particles in concentrated aerosol had a mean particle diameter of 94 nm ± 8 nm. The mean particle number count was 25 ± 14 × 104 particles/cm^3^, with a mean mass concentration of 158 ± 85 μg/m^3^.

#### UPDIABETES Study

During the 2-hour exposure periods, participants underwent a mouthpiece exposure to either freshly generated elemental carbon UFPs or filtered air, wearing a nose clip. Particle generation was done in a modified commercial generator (Palas Co., Karlsruhe, Germany); specifically, particles were generated from graphite electrodes by electric spark discharge in an argon atmosphere. This produced particles consisting of more than 95% elemental carbon, free of metals. For filtered-air exposures, air was passed through charcoal and high-efficiency particle filters and was essentially free of particles. PNC was monitored on both the inspiratory and expiratory sides of the participants. Particle median diameter was 32 nm and total PNC was 10 ± 1 × 106 particles/cm^3^.

### ECG Outcome Measurements

In the Augsburg, UPCON, and UPDIABETES studies, each participant was monitored using 12-lead Holter recorders (Mortara Instruments), while in the REHAB study, subjects underwent 3-lead (modified V2, V5, and AVF) Holter ECG recordings (Burdick Altair-DISC, Cardiac Science, Bothell, WA). All Holter recordings were annotated first automatically and then analyzed by a trained technician using custom-made programs at the University of Rochester Medical Center, which have been described previously^27^. We applied a post-processing approach to evaluate extreme values: We examined the distribution of each ECG parameter - values were considered to be outliers if they were greater than the 99^th^ percentile or smaller than the first percentile in the study population. Outliers were then further evaluated to check whether the values were valid or not comparing them to intra-lab ranges developed during a previous study^34^.

HRV (standard deviation of NN-intervals (SDNN), root mean square of successive differences (RMSSD), percentage of NN intervals longer than 50 msec (PNN50), high frequency (HF) power (0.15–0.40 Hz), low frequency (LF) power (0.04–0.15 Hz), very low frequency (VLF) power (0.0033–0.04 Hz), and total power (TP, 0–0.5 Hz)), repolarization (T-wave complexity, T-wave amplitude (Tamp), Bazett-corrected QT interval (QTc)) and other (heart rate/mean NN interval time between successive NN beats (HR/NN), deceleration capacity (DC)) parameters were determined on an hourly basis.

Factor analyses on these hourly ECG outcomes were then done separately for the four studies, which generated 4–5 factors for each study (see Rich, *et al*.^27^ for further details). We restricted the statistical analyses to those factors/representative ECG outcomes that were common to all four studies. These were: 1) SDNN (representing “overall HRV”), 2) RMSSD (representing “parasympathetic modulation”) and 3) T-wave complexity (representing “T-wave morphology”).

### Statistical analysis

#### Main analysis

We analyzed associations between hourly ECG parameters and particle metrics using additive mixed models with random participant effects. An appropriate covariance structure (first-order autocorrelation for the Augsburg panel study and compound symmetry for the Rochester studies) was included in order to account for the dependencies between repeated ECG recordings. Confounder selection for the two panel studies was done separately, however, both studies used a forward selection procedure and based the selection of confounders on model fit improvement (assessed by Akaike’s Information Criterion). As potential confounders for the analyses, we assessed long-term time trend, month of the visit, day of the week, time of day (morning versus afternoon), hour of the day, air temperature, relative humidity, barometric pressure, carbon monoxide concentration (only for REHAB panel study), and study visit number. To allow for nonlinear relationships, continuous confounders were included linearly or smoothly as penalized splines (P-splines). Confounders for the two controlled exposure studies were fixed a priori. We used the same confounder model for all ECG parameters within the same study for consistency. The specific models for each study are given in Table S1. In the Augsburg panel study, RMSSD and T-wave complexity, but not SDNN, were log-transformed to ensure normally distributed residuals. ECG parameters in the other three studies were untransformed.

For the two panel studies, we then separately added 1-hour averages of UFP, PM_2.5_, AMP, and BC concurrent with the 1-hour averages of ECG outcomes and with lags up to 6 hours to the confounder model; effects were estimated assuming a linear exposure-response relationship. For the two controlled human exposure studies, we estimated separate models for each possible combination of hour-specific UFP concentration measurement during the exposure and hour-specific endpoint measurement (i.e., outcome and UFP concentration both measured in the first hour of exposure, outcome measured in the second hour of exposure and mean UFP concentration measured over 2 hours of exposure, outcome measured in the first hour after exposure and mean UFP concentration measured over 2 hours of exposure, outcome measured in the second hour after exposure and mean UFP concentration measured over 2 hours of exposure, etc., through outcome measured in the sixth hour [lagged 1 hour to 6 hours] after exposure and mean UFP concentration measured over 2 hours of exposure). The models included responses for both particle exposure and filtered-air exposure days. As total PNC on filtered-air exposure days were essentially zero in the UPDIABETES study, we replaced all of the zero particle counts with the number 5.

Using the results from all four studies, we then applied a “discovery and replication” approach to draw conclusions about each of our research questions (Table 1). We thereby used the Augsburg panel study as the discovery panel and the Rochester REHAB panel and controlled human exposure studies as replication panels. The rationale for this procedure was that random chance can create false associations. However, because it is random, there is no reason to expect the same false associations in the different studies. We considered a research hypothesis/question to be confirmed/replicated if the same response (e.g., increased pollutant concentration associated with an adverse change in the ECG parameter) was found for both panel studies and at least one of the two controlled human exposure studies for Questions 1–3, and for both panel studies for Questions 4–6 (which could only be assessed in the panel studies).

**Table 1:** Research questions under investigation.

Question^#^	Research Hypothesis/Question
1	Are adverse changes in **total HRV** associated with increased UFP in the previous few hours?
2	Are adverse changes in **parasympathetic modulation** associated with increased UFP in the previous few hours?
3	Are adverse changes in **repolarization/T-wave morphology** associated with increased UFP in the previous few hours?
4	Are adverse changes in **total HRV** associated with increased concentrations of the other pollutants (PM_2.5_, AMP, and BC) in the previous few hours?
5	Are adverse changes in **parasympathetic modulation** associated with increased concentrations of the other pollutants (PM_2.5_, AMP, and BC) in the previous few hours?
6	Are adverse changes in **repolarization/T-wave morphology** associated with increased concentrations of the other pollutants (PM_2.5_, AMP, and BC) in the previous few hours?

We also looked at the independent effects of particle metrics by using two-pollutant models. We restricted the analyses to those pollutants having an inter-correlation <0.6, to prevent problems with collinearity. Lags with the absolute greatest single-day effect were included in these two-pollutant models. We further checked the exposure-response functions for particle metrics and ECG outcomes for deviations from linearity. Exposure-response curves were assessed using P-splines and then visual inspection to check whether the smoothed curve resembled a straight line.

We ran several sensitivity analyses to assess whether our findings were robust. A detailed description of the sensitivity analyses done for each study is given in the Supplemental Material.

## Results

Participant characteristics of each of the four studies are shown in Table 2. For a detailed description of the study populations, see Rich, *et al*.^27^. Descriptive statistics of 1-hour ECG parameters for the four studies are shown in Table 3. Early post-infarction patients of the REHAB panel study showed considerably higher 1-hour SDNN and RMSSD median values compared to those found in the other three studies.

**Table 2:** Characteristics of study populations by study.

		Augsburg Panel				Rochester REHAB		Rochester UPCON		Rochester UPDIABETES	
		Diabetes + IGT *N* = 64		Gen.Susc.* N* = 45		*N* = 73		*N* = 19		*N* = 18	
		*N*	(%)	*N*	(%)	*N*	(%)	*N*	(%)	*N*	(%)
Gender	Male	42	(66)	27	(60)	49	(67)	10	(53)	9	(50)
Age	<60 years	13	(20)	28	(62)	33	(45)	19	(100)	18	(100)
	≥60 years	51	(80)	17	(38)	40	(55)	0	(0)	0	(0)
Body mass index	<30 kg/m²	34	(53)	37	(82)	40	(55)	12	(63)	6	(33)
	≥30 kg/m²	30	(47)	8	(18)	33	(45)	7	(37)	12	(67)
Smoking	Never	26	(41)	23	(51)	34	(47)	19	(100)	0	(0)
	Former	37	(58)	18	(40)	39	(53)	0	(0)	0	(0)
	Occasional	1	(2)	4	(9)	0	(0)	0	(0)	0	(0)
Prior MI	Yes	6	(9)	0	(0)	42	(58)	0	(0)	0	(0)
Coronary heart disease	Yes	4	(6)	3	(7)	73	(100)	0	(0)	0	(0)
Hypertension	Yes	41	(64)	19	(42)	43	(59)	0	(0)	2	(11)
Diabetes	Yes	32	(50)	0	(0)	17	(23)	0	(0)	18	(100)
Anti-inflammatory medication	Yes	14	(22)	10	(22)	N/A		0	(0)	1	(6)
Corticosteroids	Yes	4	(6)	1	(2)	N/A		0	(0)	0	(0)
Statins	Yes	13	(20)	6	(13)	73	(100)	0	(0)	0	(0)
Beta blockers	Yes	19	(30)	9	(20)	66	(90)	0	(0)	3	(17)
Calcium channel blockers	Yes	8	(13)	3	(7)	7	(10)	0	(0)	1	(6)
Diuretics	Yes	25	(39)	11	(24)	20	(27)	0	(0)	1	(6)
Antithrombotic agents	Yes	14	(22)	6	(13)	N/A		0	(0)	1	(6)
Angiotensin receptor blockers	Yes	N/A		N/A		10	(14)	0	(0)	1	(6)
Angiotension-converting-enzyme inhibitor	Yes	N/A		N/A		50	(68)	0	(0)	4	(22)

Abbreviations: Gen. Susc. = participants with a genetic susceptibility; N/A = not available.

**Table 3:** Description of 1-hour ECG parameters.

	Panel	*N*	Mean	SD	Min	Q1	Median	Q3	Max
**Augsburg Panel**									
SDNN (ms)	All	2,041	80.2	28.5	11.8	60.1	77.0	97.5	198.8
	Diabetes + IGT	1,198	76.7	27.2	11.8	56.2	74.3	94.9	161.2
	Gen.Susc.	843	85.2	29.7	22.6	65.1	80.4	101.6	198.8
RMSSD (ms)	All	2,042	31.3	26.6	1.3	17.3	23.7	33.1	227.3
	Diabetes + IGT	1,198	34.0	31.8	1.3	16.6	23.0	35.7	227.3
	Gen.Susc.	844	27.5	16.2	7.2	18.3	24.5	31.8	159.8
T-wave complexity (%)	All	2,042	18.0	8.6	5.3	12.1	15.8	21.5	55.5
	Diabetes + IGT	1,198	17.5	7.5	5.6	12.2	15.9	20.7	46.0
	Gen.Susc.	844	18.8	9.9	5.3	11.9	15.6	22.9	55.5
**REHAB**									
SDNN (ms)		2,794	104.9	44.9	10.1	71.9	98.9	131.1	249.6
RMSSD (ms)		2,802	60.8	34.9	6.0	33.6	55.7	80.0	230.3
T-wave complexity (%)		2,793	7.9	8.5	0.7	3.1	5.0	9.4	66.6
**UPCON**									
SDNN (ms)		1,453	82.4	35.2	17.3	57.4	76.6	99.7	248.2
RMSSD (ms)		1,457	31.8	21.2	6.2	16.8	25.3	39.2	121.9
T-wave complexity (%)		1,432	18.1	11.5	3.8	9.3	14.6	24.1	79.1
**UPDIABETES**									
SDNN (ms)		256	75.5	27.9	21.7	57.7	71.6	89.1	207.9
RMSSD (ms)		256	34.2	20.2	7.2	19.3	28.9	44.9	130.9
T-wave complexity (%)		256	16.6	10.0	5.1	9.8	12.9	21.8	74.0

Abbreviations: SD = standard deviation; Min = minimum; Q1 = 1^st^ quartile; Q3 = 3^rd^ quartile; Max = maximum; SDNN: standard deviation of normal-to-normal (NN) beats; RMSSD: root mean square of successive differences.

Descriptive statistics and correlations of ambient particle concentrations for the Augsburg and Rochester REHAB panel studies are shown in Table 4. One-hour concentrations of all particle metrics were considerably higher in the Augsburg compared to the REHAB panel study. Descriptive statistics of total PNC during each exposure in the two controlled human exposure studies (UPCON and UPDIABETES) used in our analysis are shown in Table 5. Compared to the ambient UFP concentrations obtained in the panel studies, total PNC in UPCON and UPDIABETES were an order of magnitude higher, despite the fact that controlled exposures only lasted for two hours. Table 5 also shows the distribution of particle counts, masses, and sizes for the controlled human exposures studies. Median particle counts in the UPDIABETES study were considerably higher compared to the UPCON study.

**Table 4:** Description and correlations of 1-hour particle metrics and meteorological variables for the Augsburg Panel and Rochester REHAB Studies.

									Spearman correlation coefficients				
	*N*	Mean	SD	Min	Q1	Median	Q3	Max	UFP	AMP	BC	Temp	RH
**Augsburg Panel (March 19**, **2007**, **to December 17**, **2008)**													
PM_2.5_ (µg/m³)	15,461	13.7	11.2	0.0	5.8	10.9	18.1	106.5	0.42	0.75	0.73	−0.29	0.16
UFP (n/cm³)	14,699	9,518	6,902	937	4,892	7,629	12,049	80,858		0.70	0.58	−0.14	0.00
AMP (n/cm³)	14,699	2,060	1,535	88	1,020	1,657	2,615	17,377			0.76	−0.12	0.06
BC (µg/m³)	13,359	1.8	1.5	0.3	0.9	1.3	2.1	21.4				−0.16	0.32
Air temperature (°C)	15,398	10.8	7.9	−8.4	4.7	10.8	16.5	33.8					−0.56
Relative humidity (%)	15,398	76.9	18.3	21.0	63.3	81.3	92.8	100.0					
**Rochester REHAB (June 26**, **2006**, **to November 25**, **2009)**													
PM_2.5_ (µg/m³)	26,618	8.7	7.3	0.0	3.7	7.0	11.5	64.0	0.21	0.65	0.62	0.01	0.09
UFP (n/cm³)	29,671	4,050	3,704	12	1,931	3,183	5,136	154,980		0.56	0.38	0.00	−0.15
AMP (n/cm³)	29,671	1,041	918	0	419	790	1,374	18,838			0.65	0.18	0.01
BC (µg/m³)	26,929	0.7	0.6	−0.2	0.3	0.5	0.9	11.7				0.18	0.21
Air temperature (°C)	29,957	11.3	10.9	−17.1	2.6	12.0	19.8	37.8					−0.31
Relative humidity (%)	29,941	64.8	19.9	0.0	50.9	67.7	81.6	99.2					

Abbreviations: SD = standard deviation; Min = minimum; Q1 = 1^st^ quartile; Q3 = 3^rd^ quartile; Max = maximum; Temp = temperature; RH = relative humidity; PM_2.5_ = particulate matter with a diameter <2.5 µm; UFP = ultrafine particles (with a diameter <100 nm); AMP = accumulation mode particles (particles with a diameter between 100 nm and 500 nm); BC = black carbon.

**Table 5:** Description of controlled particle exposures in the UPCON and UPDIABETES Studies.

	N	Mean	SD	Min	Q1	Median	Q3	Max
**UPCON – 1** ^**st**^ **hour of exposure: mean total PNC** ^**b**^								
UFP (CAPS) (n/cm³)	16^a^	2.85 × 10^5^	1.80 × 10^5^	0.27 × 10^5^	1.45 × 10^5^	2.71 × 10^5^	4.12 × 10^5^	6.33 × 10^5^
Clean Air Exposure (n/cm³)	14^a^	346	769	35	58	116	254	2,996
**UPDIABETES – 1** ^**st**^ **hour of exposure: mean total PNC** ^**b**^								
UFP (EC) (n/cm³)	17^a^	9.80 × 10^6^	0.64 × 10^6^	9.13 × 10^6^	9.43 × 10^6^	9.71 × 10^6^	10.15 × 10^6^	11.57 × 10^6^
Clean Air Exposure (n/cm³)	17^a^	<5	<5	<5	<5	<5	<5	<5
**UPCON** ^**c**^ **– average of inspired particles**								
Count (n/cm³)	19	2.46 × 10^5^	1.36 × 10^5^	0.34 × 10^5^	1.40 × 10^5^	2.07 × 10^5^	3.60 × 10^5^	5.24 × 10^5^
Mass (µg/m³)	19	158	85	19	101	149	200	321
Size (nm)	19	94	8	76	87	95	99	109
**UPDIABETES** ^**d**^ **- average of inspired particles**								
Count (n/cm³)	17	9.97 × 10^6^	0.73 × 10^6^	9.16 × 10^6^	9.41 × 10^6^	9.85 × 10^6^	10.20 × 10^6^	12.06 × 10^6^
Mass (µg/m³)	17	51	3	45	49	51	52	57
Size (nm)	17	32	2	30	31	31	32	36

Abbreviations: SD = standard deviation; Min = minimum; Q1 = 1^st^ quartile; Q3 = 3^rd^ quartile; Max = maximum; CAPS = concentrated ambient particles; EC = elemental carbon.

^a^These subjects had particle count measurements for the first hour of the exposure.

^b^Mean total PNC in the 1st hour of exposure for the sample used to examine ECG outcome changes associated with the 1st hour of total PNC.

^c^*N* = 19 subjects from the UPCON Study were used in one or more health analyses (i.e., had both particle measurements and ECG recording).

^d^*N* = 17 subjects from the UPDIABETES study were used in one or more health analyses (i.e., had both particle measurements and ECG recording).

*N* = 2 subjects did not have particle measurements during an exposure.

**Figure 1:**
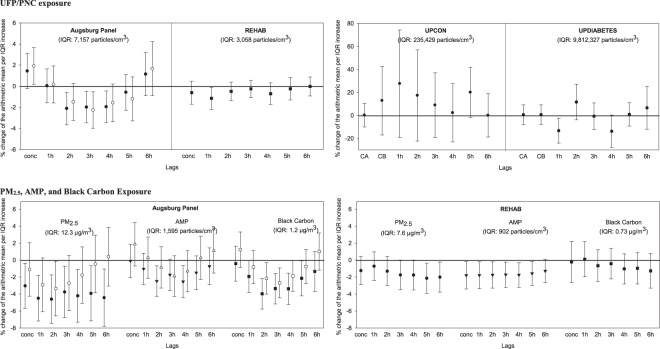
Percent change in SDNN associated with each interquartile range increase in pollutant concentration in the concurrent hour and lags 1 h to 6 h in the Augsburg Panel Study, in the Rochester REHAB Study, and in the UPCON and UPDIABETES studies. In the Augsburg Panel Study, black symbols represent individuals with type 2 diabetes or impaired glucose tolerance [Diab + IGT]; white symbols represent healthy participants with a genetic susceptibility [Gen susc]. conc = concurrent to the exposure, CA = first hour of exposure, CB = mean of 2-hour exposure, 1 h = first hour after exposure, 2 h = second hour after exposure, etc.

**Figure 2:**
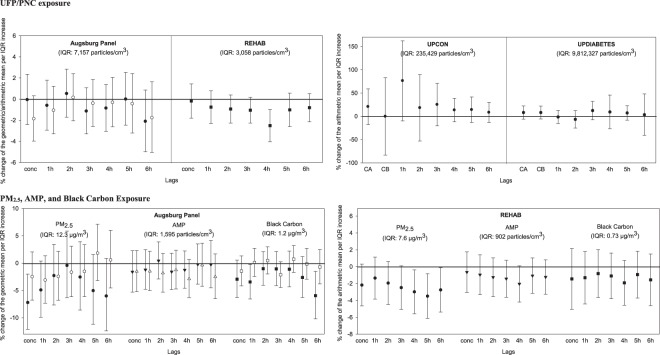
Percent change in RMSSD associated with each interquartile range increase in pollutant concentration in the concurrent hour and lags 1 h to 6 h in the Augsburg Panel Study, in the Rochester REHAB Study, and in the UPCON and UPDIABETES studies. In the Augsburg Panel Study, black symbols represent individuals with type 2 diabetes or impaired glucose tolerance [Diab + IGT]; white symbols represent healthy participants with a genetic susceptibility [Gen susc]. conc = concurrent to the exposure, CA = first hour of exposure, CB = mean of 2-hour exposure, 1 h = first hour after exposure, 2 h = second hour after exposure, etc.

**Figure 3:**
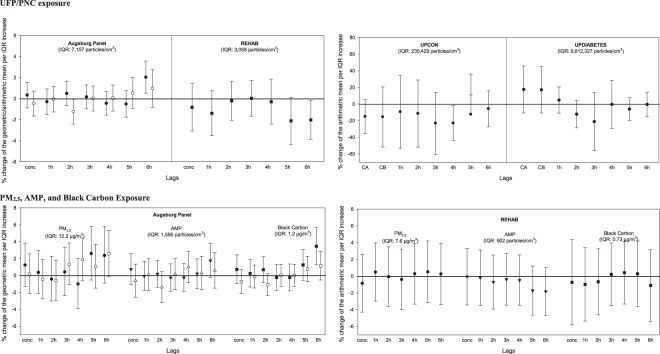
Percent change in T-wave complexity associated with each interquartile range increase in concentration in the concurrent hour and lags 1 h to 6 h in the Augsburg Panel Study, in the Rochester REHAB Study, and in the UPCON and UPDIABETES studies. In the Augsburg Panel Study, black symbols represent individuals with type 2 diabetes or impaired glucose tolerance [Diab + IGT]; white symbols represent healthy participants with a genetic susceptibility [Gen susc]. conc = concurrent to the exposure, CA = first hour of exposure, CB = mean of 2-hour exposure, 1 h = first hour after exposure, 2 h = second hour after exposure, etc.

Results for the associations between SDNN, RMSSD as well as T-wave complexity and ambient UFP as well as total PNC (research questions 1–3) are presented in Figs 1–3. Percent changes in SDNN associated with each IQR increase in ambient UFP concentrations and total PNC in the concurrent hour and at lags of 1 to 6 hours are presented in Fig. 1. For the Augsburg panel study, increased ambient UFP concentrations lagged 2 to 4 hours resulted in decreases in SDNN; the largest decrease was seen at lag 3 hours for the genetic susceptibility group (percent change [%-change]: −2.26%; 95% confidence interval [CI]: −3.98% to −0.53%) (Fig. 1 and Table S2). Similarly, in the REHAB study, we found consistent decreases in SDNN for increases in ambient UFP concentrations in the concurrent and previous five hours, with the largest effect at lag 1 hour (%-change: −1.15%; 95% CI: −2.19% to −0.11%) (Fig. 1 and Table S3). We also observed a significant decrease in SDNN associated with increased total PNC in the UPDIABETES (%-change at lag 1 hour: −13.22%; 95% CI: −24.11% to −2.33%) (Fig. 1 and Table S4), but not in the UPCON study, where increased total PNC was generally associated with non-significant increases in SDNN (Fig. 1 and Table S5). Overall, we therefore concluded that research question 1 (Table 1) was confirmed/replicated.

Figure 2 shows the percent changes in RMSSD associated with each IQR increase in ambient UFP concentrations and total PNC in the concurrent hour and at lags 1 to 6 hours. Ambient UFP was not associated with changes in RMSSD in the Augsburg panel study (Fig. 2 and Table S2), whereas in the REHAB study, we found a decreased RMSSD in association with increased UFP concentrations. The largest reduction was seen at a lag of 4 hours (%-change: −2.51%; 95% CI: −4.04% to −0.98%) (Fig. 2 and Table S3). Further, total PNC in either the UPCON (Fig. 2 and Table S4) or the UPDIABETES study (Fig. 2 and Table S5) was not associated with changes in RMSSD and therefore, we considered research question 2 (Table 1) to be not confirmed.

Percent changes in T-wave complexity associated with each IQR increase in ambient UFP concentrations and total PNC in the concurrent hour and at lags 1 to 6 hours are presented in Fig. 3. In the Augsburg panel study, increased UFP concentrations with a lag of 6 hours were associated with a 2.03%-increased T-wave complexity (95% CI: 0.52% to 3.57%) in the T2D + IGT group (Fig. 3 and Table S2). However, we observed a significant decrease in T-wave complexity in association with UFP concentrations lagged 6 hours in the REHAB study (%-change: −2.02%; 95% CI: −3.88% to −0.16%) (Fig. 3 and Table S3). In the UPCON study, total PNC were associated with decreases in T-wave complexity at all lags, with the strongest effect seen for a lag of 4 hours (%-change: −22.99%; 95% CI: −44.07% to −1.91%) (Fig. 3 and Table S4). Finally, in the UPDIABETES study, we observed decreases in T-wave complexity for total PNC lagged 2 to 6 hours; however, these effects were non-significant (Fig. 3 and Table S5). As the results of the two panel studies were contradictory, we overall concluded that research question 3 (Table 1) was not confirmed.

Results for the associations between SDNN, RMSSD as well as T-wave complexity and PM_2.5_, AMP and BC (research questions 4–6) are presented in Figs 1–3. In the Augsburg panel study, concurrent and lagged PM_2.5_ concentrations and lagged AMP and BC concentrations were all associated with (significantly) decreased SDNN (Fig. 1 and Table S2). Moreover, in the REHAB study, PM_2.5_ concentrations at lags 5 and 6 hours as well as AMP concentrations in the concurrent hour and at lags 1 hour to 5 hours were associated with 1%-2% decreases in SDNN. We found the largest SDNN reduction per an IQR increase in PM_2.5_ concentration lagged 5 hours (%-change: −2.13%; 95% CI: −3.91% to −0.35%)(Fig. 1 and Table S3). Overall, we therefore concluded that research question 4 (Table 1) was confirmed.

Further, in the Augsburg panel study, significant 3–7% decreases in RMSSD were associated with PM_2.5_ concentrations in the concurrent hour, as well as with BC lagged 1 hour and 6 hours (Fig. 2 and Table S2). Moreover, increases in PM_2.5_ concentrations at lags 4 to 6 hours were associated with a ~2.5% to 3.5% decreased RMSSD in the REHAB study (Fig. 2; Table S3). We therefore concluded that research question 5 (Table 1) was confirmed.

Finally, we observed an increased T-wave complexity per IQR increases in PM_2.5_, AMP and BC at lag 6 h in the Augsburg panel study, with the strongest effect seen for BC in the T2D/IGT group (Fig. 3; Table S2). However, in the Rochester REHAB study, there was no consistent pattern seen with any particle metrics (Fig. 3; Table S3); therefore, research question 6 (Table 1) was not confirmed.

We ran several sensitivity analyses to assess whether our findings between UFP and PM_2.5_ concentrations and SDNN were robust to additional adjustment for the other particle metric. Models including terms for both UFP and PM_2.5_ were applied to the Augsburg panel (at lags 2 and 3 hours) and the REHAB (at lags 1 and 5 hours) studies. Compared to the single-pollutant models, the effects of UFP and PM_2.5_ on SDNN generally decreased or were of similar size, and still suggestive of an effect (Table S6). In the Rochester REHAB study, changes in SDNN associated with each pollutant were generally found to be smaller or of similar size than in the single pollutant models, and also still suggestive of an effect (Table S6).

Further, we assessed the exposure-response functions of SDNN and particle metrics for deviations from linearity in the Augsburg and REHAB panel studies. In both studies, there were no deviations from linearity (data not shown).

In summary, across both panel studies, sensitivity analyses did not show results that were different from the main analysis (Tables S7 and S8). Thus, results and inference of our study could be considered robust to the modeling assumptions.

## Discussion

We observed independent associations of both UFP and PM_2.5_ concentrations and SDNN - our marker of “total HRV” - within 1 and 5 hours. Increased PM_2.5_ concentrations were also associated with decreased RMSSD, our marker of “parasympathetic modulation”. Effects of UFP and PM_2.5_ on SDNN and RMSSD generally did not differ between the various participant subgroups of our study (i.e. healthy subjects, healthy subjects with a potential genetic pre-disposition which could affect detoxifying and inflammatory pathways, individuals with diabetes or impaired glucose tolerance, and patients with acute coronary artery syndromes), although for SDNN, there was a tendency to stronger effects in individuals with diabetes or impaired glucose tolerance compared to (more) healthy participants. We did not find consistent associations of exposure to UFP and parasympathetic modulation (i.e. RMSSD) and no evidence of any effects of particle metrics on T-wave morphology (i.e. T-wave complexity).

Although we observed consistent associations between SDNN or RMSSD and particle metrics in the previous few hours, these associations were small and are likely not clinically significant. However, they provide evidence of particle-mediated subclinical physiologic changes by which air pollution may increase the risk of acute cardiovascular events^27^. When we used heart rate (or NN) as an outcome in further sensitivity analysis, results suggested that the observed effects of PM on HRV across studies were independent of associations between heart rate and air pollution (data not shown).

Many epidemiological studies have found decreased HRV in association with 24-hour mean ambient air pollution^11,12,35^, while others have reported increased HRV associated with increased air pollutant concentrations (e.g.^36–39^) or no associations at all^40,41^. Only a limited number of studies have assessed whether HRV responses to particulate air pollution occur within hours. In particular, Zanobetti, *et al*.^16^ found a −1.5% (95% CI: −2.5%; −0.4%] decrease in RMSSD per 8.2 µg/m^3^ increase in the 1-hour PM_2.5_ concentrations directly preceding ECG recording in patients with coronary artery disease in Boston, but no association with SDNN. Further, an increase of 51.8 µg/m^3^ in the prior 4-hour exposure to PM_2.5_ was associated with significant reductions in SDNN (−4.2% [95% CI: −6.4%; −1.9%]) and RMSSD (−5.5% [95% CI: −9.4%; −1.5%]) in cardiovascular disease patients in Beijing^42^. HRV parameters were also associated with individual-level PM_2.5_ exposures on a one- to six-hour basis^17^.

Further, a number of studies on taxi drivers, car commuters, cyclists or walkers have reported acute HRV changes associated with micro-environmental (e.g. cycling on high- and low traffic routes, walking along roadsides, inside vehicles) exposures to traffic-related air pollution^20–23,43^. For example, Sarnat, *et al*.^22^ conducted a study in 42 adults performing scripted highway commutes of two hours during morning rush hour in metropolitan Atlanta, Georgia. They found decreases in SDNN and RMSSD 3 hours after the commute. A study conducted in Beijing found reductions in HRV parameters of taxi drivers which were associated with increases in 30-minute concentrations of PM_2.5_ monitored inside the taxicab^43^.

We did not find any consistent effects of either ambient or controlled exposures to PM on T-wave complexity. Evidence of a relationship between elevated levels of PM and T-wave complexity is scarce^25,26^. Effect estimates of 0.84% (95% CI: 0.17%; 1.51%) in T-wave complexity in association with an IQR increase of 16.4 µg/m^3^ in the 6-hour averages of PM_2.5_ have been observed in stable ischemic heart disease patients^25^. A controlled human exposure study did not show significant associations of 2-hour exposures to UFP and markers of repolarization in healthy young subjects, although a trend toward an increased variability of T-wave complexity was observed^26^. A larger number of epidemiological studies has investigated associations between increased PM and changes in other markers of repolarization and T-wave morphology such as corrected QT-interval duration or T-wave amplitude; however, with conflicting results (e.g.^29,44–46^).

A larger number of studies have identified subgroups which are more susceptible to the harmful effects of particulate air pollution than the general population^11,12^. These studies reported that patients with pre-existing diseases such as ischemic heart disease, coronary artery disease, previous myocardial infarction, or diabetes were at an increased risk of experiencing acute exacerbation of their disease on days with high air pollution concentrations. Further, genetic factors (especially genotypes related to oxidative stress) have been shown to play a role in responsiveness to particle metrics by some studies (e.g.^34,44,47,48^). Our study participants ranged from healthy young adults to healthy individuals with a potential genetic susceptibility to oxidative injury and inflammatory pathways, patients with diabetes or impaired glucose tolerance (IGT), and patients with acute coronary artery syndromes. However, effects of UFP and PM_2.5_ on SDNN and RMSSD generally did not differ for the various participant subgroups in our study, although for SDNN, there was a tendency to stronger effects in individuals with diabetes or impaired glucose tolerance compared to (more) healthy participants.

Three biological pathways are thought to be important in the association between ambient PM and cardiovascular disease^11,12^: (1) Inhalation of particles may lead to the release of pro-inflammatory mediators or vasculoactive molecules from lung cells inducing a systemic chain reaction. This may further lead to changes in vascular function, detrimental cardiac outcomes, and induction of a pro-coagulation state (with thrombus formation, ischemic response and increase of atherosclerotic lesions; (2) Particles deposited in the pulmonary tree may be related to a perturbation of the autonomic nervous system or heart rhythm. This imbalance may be triggered by stimulating pulmonary neural reflexes or by provoking oxidative stress and subsequent inflammation in the lung. Alterations in autonomic tone can initiate cardiac arrhythmias or add to the instability of a vascular plaque; (3) UFP and PM constituents can be translocated into the blood causing endothelial dysfunction and vasoconstriction, increased blood pressure and platelet aggregation. Once in the circulation, UFP might also directly affect the heart and other organs.

A strength of our study is the use of four completed studies with data on both ambient and controlled particle exposures; moreover, all the 1-hour analysis of ECG recordings were done by the same research cardiology group, and the same basic statistical analyses were applied to both panel (Augsburg and REHAB) and controlled human exposure (UPCON and UPDIABETES) studies. However, there were also several limitations to consider. First, the controlled-exposure studies had small sample sizes; hence, there might have been limited statistical power to detect significant associations between ECG outcomes and the 2-hour exposures to UFP. However, we based conclusions on the patterns of responses across lag hours, thereby lessening the impact of this limitation^27^. We used a number of pollutants for these analyses, as different pollutants may indicate different properties of the aerosol, and also mirror different air pollution sources. It can, therefore, not be ruled out that some significant associations occurred by chance only. However, we concentrated on consistent patterns in the data which were seen across these closely correlated air pollution parameters and across different lags within the pollutants. In addition, we carefully adjusted for meteorological variables to minimize the possibility that the associations resulted from meteorological influences or seasonal differences. Further, there might have been differences in PM composition or PM sources comparing Augsburg and Rochester. However, source-apportionment studies conducted in both cities (see Rich, *et al*.^27^ for further details) suggest that sources of PM (and likely also the composition) are generally similar, although some differences were found which were mostly related to traffic-fleet composition and to the fossil fuels used to generate electricity and heat. For example, the fraction of diesel light-duty vehicles is smaller in Rochester than in Augsburg. Moreover, particle metrics used in the Augsburg and REHAB studies may vary considerably in space. Therefore, measurement error due to greater spatial variability could be present in this study as we only had measurements of one urban background station in each city to characterize exposure over an entire community. Meanwhile, there is a general agreement amongst the scientific community that single background stations which measure PM mass concentrations can be considered as representative for large urban areas, especially in panel studies where the temporal variation is assessed rather than the geographic variation^49^. Moreover, concurrent measurements of particles in the ultrafine range at different sites within one city often have shown good correlations over time despite differing magnitudes in space. This suggests that a background site might also well represent the exposure of the average population with respect to UFP if the site is carefully chosen^49,50^. To the extent that there is classical measurement error inherent, it has been shown that it is highly unlikely to bias away from the null even in the presence of covariates^51^. Therefore, measurement error in our particle metrics would likely attenuate the true association. Finally, it may be possible that there is some bias in our results, since some of the studies used had already reported associations between PM and ECG outcomes (e.g. HRV) at longer lag times (e.g. daily averages) than the hourly analyses conducted here. However, these previous analyses: 1) had not used factor analysis methods to generate functional outcome groups; 2) had not applied a discovery/replication approach to determine if both increased PM_2.5_ and UFP concentrations resulted in adverse changes in ECG outcomes (e.g. reduced HRV) across these four studies; 3) had not done such analyses across multiple different study populations (e.g. healthy, subjects with diabetes or previous myocardial infarctions), and within different air pollution mixtures, PM alone, or elemental carbon PM. Since all of these study/analysis features were substantially different from our previous analyses of these data, the potential for bias should be minimal.

## Conclusions

Even with different study designs, study populations, exposure scenarios, and exposure levels, we found consistent effects of UFP or PM_2.5_ on SDNN as well as of PM_2.5_ on RMSSD across the four studies used in this analysis. These findings thereby provided consistent evidence that recent exposures (within hours) to UFP and PM_2.5_ can induce acute pathophysiological responses.

## Supplementary information

Supplemental Material
