# Supplementary material for: Ambient and controlled exposures to particulate air pollution and acute changes in heart rate variability and repolarization
Source: Sci Rep. 2019 Feb 13;9:1946. doi: 10.1038/s41598-019-38531-9 (PMC6374365; doi:10.1038/s41598-019-38531-9)
Supplement: Supplementary file 1 — Supplemental Material [file 41598_2019_38531_MOESM1_ESM.docx]

**Supplemental Material**

## Ambient and controlled particle exposures and acute changes in heart rate variability and repolarization

Susanne Breitner^1,2^, Annette Peters^1,2^, Wojciech Zareba^3^, Regina Hampel^1^, David Oakes^3^, Jelani Wiltshire^3^, Mark W. Frampton^3^, Philip K. Hopke^4^, Josef Cyrys^1^, Mark J. Utell^3^, Cathleen Kane^3^, Alexandra Schneider^1 *^, David Q. Rich^3*^

^1^Helmholtz Zentrum München–German Research Center for Environmental Health, Neuherberg, Germany

^2^Ludwig-Maximilians-Universität München, IBE-Chair of Epidemiology, Munich, Germany

^3^University of Rochester Medical Center, Rochester, New York

^4^Clarkson University, Potsdam, New York

## ^*^Shared last authorship

**Table of contents**

**Statistical Analysis**

**Table S1 Page 2**

**Results**

**Table S2 Page 4**

**Table S3 Page 6**

**Table S4 Page 7**

**Table S5 Page 8**

**Table S6 Page 9**

**Table S7 Page 10**

**Table S8 Page 11**

**STATISTICAL ANALYSIS**

***Confounder models***

**Table S1. Confounder models for the two panel (the Augsburg panel and Rochester REHAB studies) and two con­trolled human exposure studies (UPCON and UPDIABETES).**

| Confounder | Augsburg panel | Rochester REHAB | UPCON | UPDIABETES |
| --- | --- | --- | --- | --- |
| Time trend | P-spline | -- |  |  |
| Month of visit | -- | categorical |  |  |
| Day of the week | -- | Monday, Friday, or Tuesday–Thursday |  |  |
| Time of day | morning vs. afternoon | -- |  |  |
| Hour of day | -- | categorical | categorical | categorical |
| Air temperature | 6-hour average, linear | lagged 5 hours, linear |  |  |
| Relative humidity | 6-hour average, linear | lagged 6 hours, linear |  |  |
| Carbon monoxide | -- | lagged 4 hours, linear |  |  |
| Visit number | -- | -- | 1 or 2 | 1 or 2 |

***Sensitivity analysis***

***Augsburg Panel study:*** In order to check the robustness of the effects of particle metrics, we specified different values of smoothness for the nonlinear components, especially for the time trend, or included air temperature and relative humidity with various lag hours.

As autocorrelation could be an issue in anal­yses dealing with 1-hour ECG data (which are naturally correlated with each other), we tried to check the robustness of our estimated effects using several more sen­sitivity analyses: 1) We compared the derived estimates of our a priori chosen fixed confounder model to an AIC-selected model for each outcome parameter separately; 2) we used different degrees of freedom for the trend variable to check for seasonality effects that could have an influence on autocorrelation in the data; 3) we replaced the random subject effects with fixed subject effects; 4) we assessed models with various covariance struc­tures (e.g., cs, ar[1], ar[2], ar[3], and spatial); 5) we included the lagged outcome variable (*t* − 1) in the model, which is considered to be the strictest approach for autocor­relation and might therefore result in an overadjustment, leading to estimates that are too conservative; 6) we checked the autocorrelation function (ACF) plots for the autocorrelation structure in the data.

We then compared the beta coefficients and 95% CIs from these models with our main analysis.

***Rochester REHAB Study:*** As in the Augsburg Panel study, we ran several sensitivity analyses evaluating our analytic options: 1) In addition to using the compound symmetry covariance structure, there was an additional variance term (autoregressive [1]) that estimated the variance across multiple measures within the same subject visit; 2) We only used the first hour of the subject visit in the analysis; 3) Generalized estimating equations were used to estimate the parameters of the model; 4) we mod­eled relative humidity with a linear term.

We then compared the beta coefficients and 95% CIs from these models with our main analysis described above.

**RESULTS**

**Table S2.** Percent change in ECG outcomes associated with each interquartile range increase in concurrent and 1 to 6h lagged air pollutant concentrations (taken from the stationary site) in participants in the Augsburg panel Study.

**Individuals with type-2 diabetes or impaired glucose tolerance (Diabetes + IGT).**

|  | **UFP**  **IQR = 7,157 particles/cm^3^** | | | **AMP**  **IQR = 1,595 particles/cm^3^** | | | **PM _2.5_**  **IQR = 12.3 µg/m^3^** | | | **Black Carbon**  **IQR = 1.2 µg/m^3^** | | |
| --- | --- | --- | --- | --- | --- | --- | --- | --- | --- | --- | --- | --- |
| **Pollutant**  **averaging time** | **N** | **%**  **Change** | **95% confidence**  **interval** | **N** | **%**  **Change** | **95% confidence**  **interval** | **N** | **%**  **Change** | **95% confidence**  **interval** | **N** | **% Change** | **95% confidence**  **interval** |
| **SDNN (ms)** |  |  |  |  |  |  |  |  |  |  |  |  |
| Concurrent | 1158 | 1.44%# | -0.22%, 3.10% | 1158 | -0.10% | -2.07%, 1.87% | 1185 | -3.03%* | -5.68%, -0.37% | 957 | -0.42% | -2.47%, 1.64% |
| Lag 1 hour | 1160 | 0.04% | -1.56%, 1.65% | 1160 | -1.04% | -2.89%, 0.81% | 1185 | -4.49%** | -7.18%, -1.80% | 953 | -1.93%* | -3.82%, -0.04% |
| Lag 2 hours | 1162 | -2.11%** | -3.65%, -0.58% | 1162 | -2.47%** | -4.27%, -0.68% | 1185 | -4.59%** | -7.44%, -1.75% | 954 | -3.96%** | -5.77%, -2.16% |
| Lag 3 hours | 1167 | -1.98%** | -3.47%, -0.50% | 1167 | -1.74%# | -3.58%, 0.11% | 1186 | -3.74%* | -6.77%, -0.72% | 960 | -3.36%** | -5.16%, -1.55% |
| Lag 4 hours | 1166 | -1.95%* | -3.45%, -0.45% | 1166 | -2.54%** | -4.42%, -0.65% | 1186 | -4.19%** | -7.32%, -1.07% | 966 | -3.38%** | -5.23%, -1.53% |
| Lag 5 hours | 1166 | -0.58% | -2.27%, 1.12% | 1166 | -1.49% | -3.47%, 0.50% | 1186 | -3.92%* | -7.17%, -0.67% | 974 | -2.14%* | -4.20%, -0.09% |
| Lag 6 hours | 1168 | 1.15% | -0.89%, 3.19% | 1168 | -0.73% | -2.89%, 1.44% | 1186 | -4.42%* | -7.79%, -1.05% | 980 | -1.34% | -3.68%, 1.01% |
| **RMSSD (ms)** |  |  |  |  |  |  |  |  |  |  |  |  |
| Concurrent | 1158 | -0.06% | -2.41%, 2.36% | 1158 | -1.60% | -5.32%, 2.26% | 1185 | -7.20%** | -12.11%, -2.02% | 957 | -2.95%# | -6.32%, 0.54% |
| Lag 1 hour | 1160 | -0.60% | -2.94%, 1.80% | 1160 | -1.22% | -4.79%, 2.47% | 1185 | -4.90%# | -9.90%, 0.38% | 953 | -3.45%* | -6.57%, -0.22% |
| Lag 2 hours | 1162 | 0.52% | -1.72%, 2.82% | 1162 | 0.44% | -2.89%, 3.88% | 1185 | -2.27% | -7.66%, 3.43% | 954 | -1.02% | -4.05%, 2.10% |
| Lag 3 hours | 1167 | -1.14% | -3.30%, 1.07% | 1167 | -1.58% | -4.85%, 1.80% | 1186 | -0.41% | -6.32%, 5.87% | 960 | -1.05% | -4.13%, 2.12% |
| Lag 4 hours | 1166 | -0.86% | -3.05%, 1.39% | 1166 | -1.25% | -4.61%, 2.22% | 1186 | -2.54% | -8.58%, 3.91% | 966 | -1.12% | -4.34%, 2.22% |
| Lag 5 hours | 1166 | 0.00% | -2.47%, 2.53% | 1166 | -0.24% | -3.85%, 3.51% | 1186 | -5.02% | -11.19%, 1.57% | 974 | -2.63% | -6.29%, 1.18% |
| Lag 6 hours | 1168 | -2.11% | -4.98%, 0.86% | 1168 | -0.27% | -4.51%, 4.17% | 1186 | -6.01%# | -12.40%, 0.85% | 980 | -5.95%** | -10.20%, -1.50% |
| **T-wave complexity (%)** |  |  |  |  |  |  |  |  |  |  |  |  |
| Concurrent | 1157 | 0.34% | -0.88%, 1.57% | 1157 | 0.74% | -1.07%, 2.57% | 1184 | 1.25% | -1.28%, 3.84% | 956 | 0.72% | -0.98%, 2.45% |
| Lag 1 hour | 1159 | -0.29% | -1.50%, 0.93% | 1159 | -0.06% | -1.76%, 1.67% | 1184 | 0.37% | -2.13%, 2.94% | 952 | 0.24% | -1.36%, 1.86% |
| Lag 2 hours | 1161 | 0.49% | -0.67%, 1.66% | 1161 | 0.18% | -1.41%, 1.80% | 1184 | -0.41% | -3.03%, 2.27% | 953 | 0.69% | -0.82%, 2.22% |
| Lag 3 hours | 1166 | 0.16% | -0.96%, 1.30% | 1166 | -0.26% | -1.87%, 1.38% | 1185 | 0.43% | -2.40%, 3.34% | 960 | -0.23% | -1.75%, 1.32% |
| Lag 4 hours | 1165 | -0.44% | -1.57%, 0.70% | 1165 | -0.24% | -1.88%, 1.43% | 1185 | -0.99% | -3.89%, 2.00% | 965 | -0.24% | -1.81%, 1.36% |
| Lag 5 hours | 1165 | -0.51% | -1.76%, 0.76% | 1165 | 0.22% | -1.53%, 1.99% | 1185 | 2.60% | -0.52%, 5.83% | 973 | 1.25% | -0.55%, 3.09% |
| Lag 6 hours | 1167 | 2.03% ** | 0.52%, 3.57% | 1167 | 1.77%# | -0.23%, 3.81%# | 1185 | 2.38% | -0.90%, 5.76% | 979 | 3.46%** | 1.29%, 5.69% |

**Individuals with potential genetic predisposition on the detoxifying and inflammatory pathways (Gen susc).**

|  | **UFP**  **IQR = 7,157 particles/cm^3^** | | | **AMP**  **IQR = 1,595 particles/cm^3^** | | | **PM _2.5_**  **IQR = 12.3 µg/m^3^** | | | | **Black Carbon**  **IQR = 1.2 µg/m^3^** | | | |  |
| --- | --- | --- | --- | --- | --- | --- | --- | --- | --- | --- | --- | --- | --- | --- | --- |
| **Pollutant**  **averaging time** | **N** | **%**  **Change** | **95% confidence**  **interval** | **N** | **%**  **Change** | **95% confidence**  **interval** | | **N** | **%**  **Change** | **95% confidence**  **interval** | **N** | **% Change** | **95% confidence**  **interval** |  |  |
| **SDNN (ms)** |  |  |  |  |  |  | |  |  |  |  |  |  |  |  |
| Concurrent | 782 | 1.92%* | 0.19%, 3.65% | 782 | 1.88% | -0.69%, 4.44% | 823 | | -1.10% | -4.25%, 2.05% | 796 | 1.23% | -0.85%, 3.32% | | |
| Lag 1 hour | 778 | 0.17% | -1.59%, 1.92% | 778 | 0.29% | -2.14%, 2.72% | 823 | | -2.92%# | -6.08%, 0.25% | 794 | -0.81% | -2.78%, 1.15% | | |
| Lag 2 hours | 772 | -1.49%# | -3.24%, 0.26% | 772 | -0.85% | -3.28%, 1.58% | 823 | | -3.34%* | -6.57%, -0.12% | 794 | -2.15%* | -4.01%, -0.29% | | |
| Lag 3 hours | 764 | -2.26%* | -3.98%, -0.53% | 764 | -1.89% | -4.29%, 0.51% | 823 | | -2.72% | -5.97%, 0.54% | 796 | -2.69%** | -4.45%, -0.92% | | |
| Lag 4 hours | 756 | -1.57%# | -3.35%, 0.22% | 756 | -1.31% | -3.72%, 1.11% | 823 | | -1.76% | -5.08%, 1.55% | 803 | -1.87%* | -3.68%, -0.06% | | |
| Lag 5 hours | 753 | -1.21% | -3.29%, 0.88% | 753 | 0.27% | -2.28%, 2.82% | 823 | | -0.46% | -3.86%, 2.94% | 804 | -0.76% | -2.77%, 1.25% | | |
| Lag 6 hours | 752 | 1.66% | -0.90%, 4.22% | 752 | 1.12% | -1.53%, 3.77% | 823 | | 0.41% | 3.05%, 3.88% | 811 | 1.01% | -1.19%, 3.20% | | |
| **RMSSD (ms)** |  |  |  |  |  |  |  | |  |  |  |  |  | | |
| Concurrent | 783 | -1.86% | -3.98%, 0.31%# | 783 | -1.53% | -5.21%, 2.30% | 824 | | -2.47% | -6.78%, 2.04% | 797 | -1.46% | -4.17%, 1.31% | | |
| Lag 1 hour | 779 | -1.06% | -3.28%, 1.21% | 779 | -1.50% | -5.03%, 2.17% | 824 | | -3.09% | -7.33%, 1.35% | 795 | 0.10% | -2.45%, 2.71% | | |
| Lag 2 hours | 773 | 0.15% | -2.05%, 2.40% | 773 | -1.83% | -5.31%, 1.77% | 824 | | -2.42% | -6.80%, 2.16% | 795 | 0.47% | -1.98%, 2.98% | | |
| Lag 3 hours | 765 | -0.40% | -2.59%, 1.85% | 765 | -1.25% | -4.73%, 2.35% | 824 | | -1.65% | -6.16%, 3.08% | 797 | -2.13%# | -4.46%, 0.26% | | |
| Lag 4 hours | 757 | -0.31% | -2.62%, 2.05% | 757 | -2.89% | -6.33%, 0.68% | 824 | | -1.50% | -6.15%, 3.37% | 804 | 0.72% | -1.71%, 3.21% | | |
| Lag 5 hours | 754 | -0.44% | -3.20%, 2.41% | 754 | -0.38% | -4.24%, 3.65% | 824 | | 1.83% | -3.19%, 7.11% | 806 | -0.15% | -2.93%, 2.71% | | |
| Lag 6 hours | 753 | -1.76% | -5.05%, 1.64% | 753 | -2.51% | -6.51%, 1.68% | 824 | | 0.59% | -4.54%, 6.01% | 812 | -0.70% | -3.79%, 2.49% | | |
| **T-wave complexity (%)** |  |  |  |  |  |  |  | |  |  |  |  |  | | |
| Concurrent | 783 | -0.47% | -1.64%, 0.71% | 783 | -0.63% | -2.57%, 1.35% | 824 | | 0.19% | -2.13%, 2.56% | 797 | -0.72% | -2.17%. 0.74% | | |
| Lag 1 hour | 779 | -0.06% | -1.27%, 1.17% | 779 | 0.09% | -1.78%, 1.99% | 824 | | -0.46% | -2.76%, 1.89% | 795 | -0.13% | -1.48%. 1.24% | | |
| Lag 2 hours | 773 | -1.24%* | -2.41%, -0.06% | 773 | -1.39% | -3.20%, 0.45% | 824 | | -0.59% | -2.94%, 1.82% | 795 | -1.09% | -2.38%. 0.22% | | |
| Lag 3 hours | 765 | 0.01% | -1.17%, 1.21% | 765 | 0.17% | -1.66%, 2.02% | 824 | | 1.35% | -1.08%, 3.84% | 797 | 0.02% | -1.26%. 1.32% | | |
| Lag 4 hours | 757 | 0.05% | -1.18%, 1.29% | 757 | 1.00% | -0.83%, 2.87% | 824 | | 1.89% | -0.61%, 4.46% | 804 | 0.01% | -1.29%. 1.33% | | |
| Lag 5 hours | 754 | 0.52% | -0.95%, 2.01% | 754 | 0.28% | -1.68%, 2.28% | 824 | | 1.08% | -1.48%, 3.71% | 806 | 0.76% | -0.74%. 2.28% | | |
| Lag 6 hours | 753 | 0.96% | -0.83%, 2.79% | 753 | 0.59% | -1.49%, 2.72% | 824 | | 2.58%# | -0.11%, 5.34% | 812 | 1.14% | -0.53%. 2.84% | | |

# p<0.10

* p<0.05

**p<0.01

**Table S3.** Percent change in outcomes associated with each interquartile range increase in concurrent and 1 to 6h lagged air pollutant concentrations in participants in the REHAB Study.

|  | **UFP**  **IQR = 3,058 particles/cm^3^** | | | | | | | | | | | | **AMP**  **IQR = 902 particles/cm^3^** | | | | | | | | | | | | **PM _2.5_**  **IQR = 7.6 µg/m^3^** | | | | | | | | | | | | **Black Carbon**  **IQR = 0.73 µg/m^3^** | | | | | | | | | | | |  |  |  |
| --- | --- | --- | --- | --- | --- | --- | --- | --- | --- | --- | --- | --- | --- | --- | --- | --- | --- | --- | --- | --- | --- | --- | --- | --- | --- | --- | --- | --- | --- | --- | --- | --- | --- | --- | --- | --- | --- | --- | --- | --- | --- | --- | --- | --- | --- | --- | --- | --- | --- | --- | --- |
|  | **N** | | | | **%**  **Change** | | | | **95% CI** | | | | **N** | | | | **%**  **Change** | | | | **95% CI** | | | | **N** | | | | **%**  **Change** | | | | **95% CI** | | | | **N** | | | | **% Change** | | | | **95% CI** | | | |  |  |  |
| **SDNN**  **(ms)** |  | | | |  | | | |  | | | |  | | | |  | | | |  | | | |  | | | |  | | | |  | | | |  | | | |  | | | |  | | | |  |  |  |
| Concurrent | | | | 2536 | | | | -0.61% | | | | -1.70%,0.47% | | | | 2536 | | | | -1.79%* | | | | -3.41%,-0.17% | | | | 2251 | | | | -1.23% | | | | -2.89%,0.44% | | | | 2326 | | | | -0.21% | | | | -2.64%, 2.21% | | | |
| Lag 1 hour | | | | 2536 | | | | -1.15%* | | | | -2.19%,-0.11% | | | | 2536 | | | | -1.78%* | | | | -3.37%,-0.19% | | | | 2259 | | | | -0.71% | | | | -2.39%,0.97% | | | | 2314 | | | | 0.10% | | | | -1.97%, 2.17% | | | |
| Lag 2 hours | | | | 2537 | | | | -0.49% | | | | -1.38%,0.40% | | | | 2537 | | | | -1.75%* | | | | -3.30%,-0.20% | | | | 2265 | | | | -1.30% | | | | -3.01%,0.42% | | | | 2302 | | | | -0.66% | | | | -2.55%, 1.22% | | | |
| Lag 3 hours | | | | 2539 | | | | -0.25% | | | | -1.07%,0.56% | | | | 2539 | | | | -1.71%* | | | | -3.19%,-0.24% | | | | 2271 | | | | -1.74%# | | | | -3.49%,0.00% | | | | 2298 | | | | -0.42% | | | | -2.22%, 1.38% | | | |
| Lag 4 hours | | | | 2540 | | | | -0.71% | | | | -1.73%,0.32% | | | | 2540 | | | | -1.75%* | | | | -3.19%,-0.31% | | | | 2275 | | | | -1.75%# | | | | -3.51%,0.00% | | | | 2296 | | | | -1.03% | | | | -2.81%, 0.74% | | | |
| Lag 5 hours | | | | 2540 | | | | -0.24% | | | | -1.30%,0.83% | | | | 2540 | | | | -1.57%* | | | | -2.97%,-0.16% | | | | 2280 | | | | -2.13%* | | | | -3.91%,-0.35% | | | | 2293 | | | | -0.96% | | | | -2.81%, 0.89% | | | |
| Lag 6 hours | | | | 2540 | | | | -0.02% | | | | -0.92%,0.87% | | | | 2540 | | | | -1.28%# | | | | -2.66%,0.09% | | | | 2283 | | | | -2.00%* | | | | -3.77%,-0.23% | | | | 2289 | | | | -1.27% | | | | -3.30%, 0.76% | | | |
| **RMSSD (ms)** |  | | | |  | | | |  | | | |  | | | |  | | | |  | | | |  | | | |  | | | |  | | | |  | | | |  | | | |  | | | |  |  |  |
| Concurrent | | | 2545 | | | | -0.18% | | | | -1.80%,1.44% | | | | 2545 | | | | -0.65% | | | | -3.06%,1.76% | | | | 2260 | | | | -2.18%# | | | | -4.65%,0.30% | | | | 2336 | | | | -1.45% | | | | -5.05%, 2.15% | | | |  |
| Lag 1 hour | | | 2545 | | | | -0.76% | | | | -2.31%,0.79% | | | | 2545 | | | | -0.96% | | | | -3.32%,1.40% | | | | 2268 | | | | -1.35% | | | | -3.84%,1.14% | | | | 2324 | | | | -1.31% | | | | -4.42%, 1.80% | | | |  |
| Lag 2 hours | | | 2546 | | | | -0.94% | | | | -2.27%,0.39% | | | | 2546 | | | | -1.26% | | | | -3.56%,1.05% | | | | 2274 | | | | -1.94% | | | | -4.48%,0.61% | | | | 2312 | | | | -0.82% | | | | -3.65%, 2.02% | | | |  |
| Lag 3 hours | | | 2548 | | | | -1.05%# | | | | -2.27%,0.17% | | | | 2548 | | | | -1.43% | | | | -3.62%,0.76% | | | | 2280 | | | | -2.48%# | | | | -5.06%,0.10% | | | | 2308 | | | | -1.10% | | | | -3.81%, 1.62% | | | |  |
| Lag 4 hours | | | 2549 | | | | -2.51%** | | | | -4.04%,-0.98% | | | | 2549 | | | | -2.04%# | | | | -4.19%,0.10% | | | | 2284 | | | | -2.98%* | | | | -5.58%,-0.38% | | | | 2306 | | | | -1.92% | | | | -4.58%, 0.74% | | | |  |
| Lag 5 hours | | | 2549 | | | | -1.02% | | | | -2.60%,0.56% | | | | 2549 | | | | -1.07% | | | | -3.16%,1.03% | | | | 2289 | | | | -3.49%** | | | | -6.13%,-0.84% | | | | 2303 | | | | -0.94% | | | | -3.72%, 1.83% | | | |  |
| Lag 6 hours | | | 2549 | | | | -0.81% | | | | -2.15%,0.52% | | | | 2549 | | | | -1.23% | | | | -3.27%,0.81% | | | | 2292 | | | | -2.75%* | | | | -5.38%,-0.12% | | | | 2299 | | | | -1.56% | | | | -4.62%, 1.50% | | | |  |
| **T-wave complexity (%)** | |  | | | |  | | | |  | | | |  | | | |  | | | |  | | | |  | | | |  | | | |  | | | |  | | | |  | | | |  | | | |  |  |
| Concurrent | | | 2565 | | | | -0.82% | | | | -3.07%, 1.42% | | | | 2565 | | | | -0.06% | | | | -3.43%,3.31% | | | | 2286 | | | | -0.84% | | | | -4.28%,2.60% | | | | 2354 | | | | -0.73% | | | | -5.80%,4.35% | | | |  |
| Lag 1 hour | | | 2565 | | | | -1.39% | | | | -3.53%, 0.75% | | | | 2565 | | | | -0.18% | | | | -3.49%,3.13% | | | | 2293 | | | | 0.50% | | | | -2.97%,3.98% | | | | 2343 | | | | -0.98% | | | | -5.36%,3.39% | | | |  |
| Lag 2 hours | | | 2566 | | | | -0.20% | | | | -2.05%, 1.65% | | | | 2566 | | | | -0.70% | | | | -3.92%,2.53% | | | | 2299 | | | | -0.05% | | | | -3.59%,3.50% | | | | 2332 | | | | -0.66% | | | | -4.60%,3.29% | | | |  |
| Lag 3 hours | | | 2568 | | | | 0.03% | | | | -1.67%, 1.73% | | | | 2568 | | | | -0.37% | | | | -3.46%,2.72% | | | | 2304 | | | | -0.38% | | | | -3.99%,3.24% | | | | 2327 | | | | 0.22% | | | | -3.52%,3.96% | | | |  |
| Lag 4 hours | | | 2569 | | | | -0.29% | | | | -2.42%, 1.85% | | | | 2569 | | | | -0.47% | | | | -3.47%,2.54% | | | | 2308 | | | | 0.30% | | | | -3.33%,3.92% | | | | 2325 | | | | 0.42% | | | | -3.31%,4.15% | | | |  |
| Lag 5 hours | | | 2569 | | | | -2.11%# | | | | -4.32%, 0.10% | | | | 2569 | | | | -1.71% | | | | -4.65%,1.22% | | | | 2313 | | | | 0.53% | | | | -3.15%,4.22% | | | | 2323 | | | | 0.29% | | | | -3.63%,4.21% | | | |  |
| Lag 6 hours | | | 2569 | | | | -2.02%* | | | | -3.88%, -0.16% | | | | 2569 | | | | -1.82% | | | | -4.68%,1.05% | | | | 2316 | | | | 0.27% | | | | -3.37%,3.91% | | | | 2319 | | | | -1.10% | | | | -5.40%,3.19% | | | |  |

# p<0.10

* p<0.05

**p<0.01

**Table S4.** Percent change in hourly ECG outcomes, associated with each 235,429 particles/cm^3^ in total particle count in the UPCON Study.

| **Time of Total Particle Count Exposure** | **Time of Holter Monitor Recording** | **N** | **%**  **Change** | **95% CI** |
| --- | --- | --- | --- | --- |
| **SDNN (ms)** |  |  |  |  |
| Hour 1 | Hour 1 of Exposure | 30 | 0.16% | -10.09%,10.41% |
| Hours 1 & 2 | Hour 2 of Exposure | 30 | 12.90% | -17.04%,42.84% |
| Hours 1 & 2 | Lag 1h | 30 | 27.62% | -19.07%,74.32% |
| Hours 1 & 2 | Lag 2h | 28 | 17.34% | -22.36%,57.05% |
| Hours 1 & 2 | Lag 3h | 31 | 8.93% | -19.28%,37.15% |
| Hours 1 & 2 | Lag 4h | 32 | 2.28% | -23.02%,27.58% |
| Hours 1 & 2 | Lag 5h | 32 | 20.06%# | -1.63%,41.75% |
| Hours 1 & 2 | Lag 6h | 33 | 0.01% | -18.78%,18.81% |
| **RMSSD (ms)** |  |  |  |  |
| Hour 1 | Hour 1 of Exposure | 30 | 20.58% | -17.69%,58.85% |
| Hours 1 & 2 | Hour 2 of Exposure | 30 | -0.34% | -83.47%,82.79% |
| Hours 1 & 2 | Lag 1h | 30 | 76.22%# | -9.75%,162.18% |
| Hours 1 & 2 | Lag 2h | 28 | 18.18% | -53.11%,89.47% |
| Hours 1 & 2 | Lag 3h | 31 | 25.10% | -20.65%,70.86% |
| Hours 1 & 2 | Lag 4h | 32 | 13.28% | -11.85%,38.40% |
| Hours 1 & 2 | Lag 5h | 32 | 14.07% | -13.29%,41.42% |
| Hours 1 & 2 | Lag 6h | 33 | 8.24% | -13.52%,30.01% |
| **T-wave complexity (%)** | |  |  |  |
| Hour 1 | Hour 1 of Exposure | 30 | -15.12% | -35.49%,5.26% |
| Hours 1 & 2 | Hour 2 of Exposure | 30 | -15.52% | -51.69%,20.65% |
| Hours 1 & 2 | Lag 1h | 30 | -9.47% | -53.00%,34.06% |
| Hours 1 & 2 | Lag 2h | 28 | -11.44% | -51.88%,29.00% |
| Hours 1 & 2 | Lag 3h | 31 | -23.14% | -60.41%,14.12% |
| Hours 1 & 2 | Lag 4h | 31 | -22.99%* | -44.07%,-1.91% |
| Hours 1 & 2 | Lag 5h | 31 | -12.47% | -35.96%,11.03% |
| Hours 1 & 2 | Lag 6h | 33 | -5.75% | -27.50%,16.00% |

# p<0.10; * p<0.05; **p<0.01

**Table S5.** Percent change in hourly ECG outcomes, associated with each 9,812,327 p/cm^3^ in 2 hour mean total particle count in the UPDIABETES Study.

| **Time of Total Particle Count Exposure** | **Time of Holter Monitor Recording** | **N** | **%**  **Change** | **95% CI** |
| --- | --- | --- | --- | --- |
| **SDNN (ms)** |  |  |  |  |
| Hour 1 | Hour 1 of Exposure | 34 | 0.60% | -8.02%,9.22% |
| Hours 1 & 2 | Hour 2 of Exposure | 34 | 0.69% | -7.82%,9.20% |
| Hours 1 & 2 | Lag 1h | 34 | -13.22%* | -24.11%,-2.33% |
| Hours 1 & 2 | Lag 2h | 33 | 11.67% | -3.84%,27.17% |
| Hours 1 & 2 | Lag 3h | 33 | -0.65% | -12.15%,10.85% |
| Hours 1 & 2 | Lag 4h | 33 | -13.75%# | -27.75%,0.25% |
| Hours 1 & 2 | Lag 5h | 33 | 0.93% | -9.23%,11.08% |
| Hours 1 & 2 | Lag 6h | 22 | 6.66% | -11.98%,25.29% |
| **RMSSD (ms)** |  |  |  |  |
| Hour 1 | Hour 1 of Exposure | 34 | 8.11% | -6.06%,22.27% |
| Hours 1 & 2 | Hour 2 of Exposure | 34 | 8.12% | -5.86%,22.10% |
| Hours 1 & 2 | Lag 1h | 34 | -1.44% | -15.58%,12.71% |
| Hours 1 & 2 | Lag 2h | 33 | -6.52% | -25.36%,12.32% |
| Hours 1 & 2 | Lag 3h | 33 | 12.36% | -7.50%,32.21% |
| Hours 1 & 2 | Lag 4h | 33 | 9.32% | -26.92%,45.55% |
| Hours 1 & 2 | Lag 5h | 33 | 7.19% | -8.32%,22.70% |
| Hours 1 & 2 | Lag 6h | 22 | 3.42% | -41.08%,47.92% |
| **T-wave complexity (%)** | | |  |  |
| Hour 1 | Hour 1 of Exposure | 34 | 17.59% | -10.63%,45.80% |
| Hours 1 & 2 | Hour 2 of Exposure | 34 | 17.21% | -10.70%,45.11% |
| Hours 1 & 2 | Lag 1h | 34 | 4.81% | -10.95%,20.57% |
| Hours 1 & 2 | Lag 2h | 33 | -12.14% | -28.70%,4.43% |
| Hours 1 & 2 | Lag 3h | 33 | -21.06% | -56.01%,13.89% |
| Hours 1 & 2 | Lag 4h | 33 | -0.32% | -29.24%,28.61% |
| Hours 1 & 2 | Lag 5h | 33 | -6.03% | -19.53%,7.47% |
| Hours 1 & 2 | Lag 6h | 22 | -0.44% | -15.19%,14.31% |

# p<0.10

* p<0.05

**p<0.01

**Table S6.** Two-pollutant models: Percent change in SDNN (ms) associated with each interquartile range increase in concurrent and 1h to 6h lagged UFP and PM_2.5_ concentrations in participants in the Augsburg Panel Study and Rochester REHAB Study, by study and study group.

1. **Augsburg Panel Study**

|  |  | **UFP**  **IQR = 7,157 particles/cm^3^** | | **PM _2.5_**  **IQR = 12.3 µg/m^3^** | |
| --- | --- | --- | --- | --- | --- |
| **Group** | **Pollutant**  **averaging time** | **%**  **Change** | **95% confidence**  **interval** | **%**  **Change** | **95% confidence**  **interval** |
|  |  |  |  |  |  |
| Diabetes + IGT group | Lag 2h (60-119min) | -1.23% | (-2.96%, 0.51%) | -3.45% | (-6.73%, -0.17%)* |
|  | Lag 3h (120-179min) | -1.48% | (-3.18%, 0.21%)# | -2.12% | (-5.63%, 1.40%) |
|  |  |  |  |  |  |
| Genetic Susceptibility | Lag 2h (60-119min) | -0.99% | (-2.88%, 0.90%) | -2.49% | (-6.13%, 1.15%) |
|  | Lag 3h (120-179min) | 1.96% | (-3.85%, -0.08%)* | -1.38% | (-5.10%, 2.35%) |

Spearman correlation between UFP and PM_2.5_: r=0.42; Spearman correlation between UFP and Black Carbon: r=0.58

1. **ROCHESTER REHAB STUDY**

|  |  | **UFP**  **IQR = 1980 particles/cm^3^** | | **AMP**  **IQR = 902 particles/cm^3^** | | **PM _2.5_**  **IQR = 7.6 µg/m^3^** | |
| --- | --- | --- | --- | --- | --- | --- | --- |
| **Pollutant**  **averaging time** | **N** | **%**  **Change** | **95% confidence**  **interval** | **%**  **Change** | **95% confidence**  **interval** | **%**  **Change** | **95% confidence**  **interval** |
|  |  |  |  |  |  |  |  |
| Lag 1h | 2536 | -0.85% | -1.96%,0.26% | -1.33% | -3.02%,0.37% | -- | -- |
| Lag 1h | 2244 | -0.78% | -1.94%,0.37% | -- | -- | -0.60% | -2.30%,1.10% |
| Lag 1h | 2244 | -- | -- | -2.10%# | -4.29%,0.09% | 0.58% | -1.58%,2.73% |
| Lag 5h | 2540 | 0.32% | -0.85%,1.48% | -1.74%* | -3.29%,-0.19% | -- | -- |
| Lag 5h | 2272 | -0.45% | -1.85%,0.94% | -- | **--** | -2.11%* | -3.91%,-0.31% |
| Lag 5h | 2272 | -- | -- | -0.99% | -2.83%,0.86% | -1.56% | -3.69%,0.57% |

Spearman correlation between UFP and AMP: r=0.60; Spearman correlation between UFP and PM_2.5_: r=0.16.

# p<0.10

* p<0.05

**p<0.01

**Table S7.** Sensitivity Analyses – Percent change in SDNN associated with each interquartile range increase in 2h to 3h lagged air pollutant concentrations (IQRs for UFP: 7,157 particles/cm^3^; PM_2.5_: 12.3 µg/m^3^; BC 1.2 µg/m^3^) in participants of the Augsburg Panel Study.

1. Individuals with type-2 diabetes or impaired glucose tolerance (Diab + IGT)

| **Pollutant and Lag** | **Main Model**  **Fixed set of confounders, trend smooth, and AR(1) as covariance structure** | | **Model #1**  **Confounders chosen by AIC** | | **Model #2**  **Trend as polynomial of 4^th^ degree** | | **Model #3**  **Fixed participant effect** | | **Model #4**  **Compound Symmetry as covariance structure** | | **Model #5**  **Lagged outcome and Compound Symmetry as covariance structure** | |
| --- | --- | --- | --- | --- | --- | --- | --- | --- | --- | --- | --- | --- |
|  | **%**  **Change** | **95% confidence**  **interval** | **%**  **Change** | **95% confidence**  **interval** | **%**  **Change** | **95% confidence**  **interval** | **%**  **Change** | **95% confidence**  **interval** | **%**  **Change** | **95% confidence**  **interval** | **%**  **Change** | **95% confidence**  **interval** |
| **UFP** Lag 2h | -2.11 | -3.65,-0.58** | -1.93 | -3.42,-0.43* | -2.18 | -3.71,-0.64** | -2.11 | -3.55,-0.66** | -2.33 | -3.88,-0.78** | -1.28 | -2.81,0.25 |
| **PM_2.5_** Lag 2h | -4.59 | -7.44,-1.75** | -4.33 | -7.06,-1.59** | -4.77 | -7.64,-1.90** | -5.00 | -7.67,-2.33** | -4.64 | -7.62,-1.66** | -3.09 | -5.82,-0.37* |
| **BC** Lag 2h | -3.97 | -5.77,-2.16** | -3.73 | -5.48,-1.98** | -3.96 | -5.77,-2.15** | -4.07 | -5.75,-2.39** | -4.16 | -6.02,-2.29** | -2.50 | -4.36,-0.64** |

1. Individuals with potential genetic predisposition (Gen susc)

| **Pollutant and Lag** | **Main Model**  **Fixed set of confounders, trend smooth, and AR(1) as covariance structure** | | **Model #1**  **Confounders chosen by AIC** | | **Model #2**  **Trend as polynomial of 4^th^ degree** | | **Model #3**  **Fixed participant effect** | | **Model #4**  **Compound Symmetry as covariance structure** | | **Model #5**  **Lagged outcome and Compound Symmetry as covariance structure** | |
| --- | --- | --- | --- | --- | --- | --- | --- | --- | --- | --- | --- | --- |
|  | **%**  **Change** | **95% confidence**  **interval** | **%**  **Change** | **95% confidence**  **interval** | **%**  **Change** | **95% confidence**  **interval** | **%**  **Change** | **95% confidence**  **interval** | **%**  **Change** | **95% confidence**  **interval** | **%**  **Change** | **95% confidence**  **interval** |
| **UFP** Lag 3h | -2.26 | -3.98,-0.53* | -1.71 | -3.45,0.03# | -2.34 | -4.07,-0.61** | -2.16 | -3.76,-0.55** | -2.71 | -4.36,-1.06** | 0.28 | -1.12,1.68 |
| **PM_2.5_** Lag 2h | -3.34 | -6.57,-0.12* | -3.04 | -6.10,0.01# | -3.24 | -6.49,0.01# | -3.00 | -6.03,0.04# | -3.73 | -6.89,-0.56* | -1.07 | -3.59,1.45 |
| **BC** Lag 3h | -2.69 | -4.45,-0.92** | -2.01 | -3.76,-0.26* | -2.90 | -4.72,-1.08** | -2.53 | -4.19,-0.89** | -3.24 | -4.96,-1.52** | -0.52 | -1.98,0.94 |

# p<0.10; * p<0.05; **p<0.01

**Table S8.** Percent change in SDNN associated with each interquartile range increase in the concurrent hour and 1h to 6h lagged air pollutant concentrations in participants in the REHAB Study for different model specifications.

| **Pollutant and Lag** | **SA Model #1** | | **SA Model #2** | | **SA Model #3** | | **SA Model #4** | | **Main Analysis** | |
| --- | --- | --- | --- | --- | --- | --- | --- | --- | --- | --- |
|  | **β** | **95% CI** | **β** | **95% CI** | **β** | **95% CI** | **β** | **95% CI** | **β** | **95% CI** |
| **UFP** |  |  |  |  |  |  |  |  |  |  |
| Concurrent | -0.29 | -1.29,0.71 | -1.06 | -2.38,0.26 | -0.48 | -1.42,0.46 | -0.46 | -1.56,0.65 | -0.61 | -1.70,0.47 |
| Lag 1h | -0.89# | -1.86,0.09 | -2.63** | -4.02,-1.23 | -1.22** | -2.10,-0.33 | -1.15* | -2.21,-0.09 | -1.15* | -2.19,-0.11 |
| Lag 2h | -0.68 | -1.51,0.14 | -1.09* | -2.09,-0.09 | -0.65 | -1.46,0.16 | -0.62 | -1.52,0.28 | -0.49 | -1.38,0.40 |
| Lag 3h | -0.66# | -1.45,0.12 | -1.24* | -2.37,-0.12 | -0.44 | -1.11,0.23 | -0.41 | -1.22,0.40 | -0.25 | -1.07,0.56 |
| Lag 4h | -1.28** | -2.26,-0.31 | -1.80* | -3.57,-0.04 | -0.87** | -1.50,-0.25 | -0.83# | -1.76,0.11 | -0.71 | -1.73,0.32 |
| Lag 5h | -0.62 | -1.65,0.40 | -0.25 | -1.43,0.92 | -0.41 | -1.13,0.30 | -0.39 | -1.47,0.69 | -0.24 | -1.30,0.83 |
| Lag 6h | -0.11 | -0.98,0.77 | -0.04 | -1.20,1.12 | -0.04 | -0.79,0.71 | -0.04 | -0.95,0.88 | -0.02 | -0.92,0.87 |
| **AMP** |  |  |  |  |  |  |  |  |  |  |
| Concurrent | -1.49* | -2.90,-0.07 | -3.12** | -5.17,-1.07 | -2.03# | -4.14,0.08 | -1.95* | -3.59,-0.32 | -1.79* | -3.41,-0.17 |
| Lag 1h | -1.65* | -3.04,-0.27 | -3.49** | -5.53,-1.45 | -2.09* | -3.99,-0.19 | -2.01* | -3.62,-0.41 | -1.78* | -3.37,-0.19 |
| Lag 2h | -1.77** | -3.12,-0.42 | -3.50** | -5.44,-1.56 | -2.22* | -3.99,-0.45 | -2.14** | -3.69,-0.58 | -1.75* | -3.30,-0.20 |
| Lag 3h | -1.86** | -3.16,-0.57 | -3.62** | -5.47,-1.77 | -2.12** | -3.63,-0.60 | -2.04** | -3.52,-0.55 | -1.71* | -3.19,-0.24 |
| Lag 4h | -1.81** | -3.08,-0.54 | -3.14** | -4.99,-1.29 | -2.15** | -3.62,-0.69 | -2.07** | -3.51,-0.62 | -1.75* | -3.19,-0.31 |
| Lag 5h | -1.59* | -2.83,-0.35 | -2.69** | -4.45,-0.92 | -2.00** | -3.18,-0.82 | -1.92** | -3.34,-0.51 | -1.57* | -2.97,-0.16 |
| Lag 6h | -1.32* | -2.53,-0.10 | -2.27* | -4.03,-0.51 | -1.69** | -2.82,-0.55 | -1.62* | -3.01,-0.23 | -1.28# | -2.66,0.09 |
| **PM_2.5_** | |  |  |  |  |  |  |  |  |  |
| Concurrent | -0.89 | -2.34,0.57 | -1.00 | -3.15,1.16 | -1.35 | -3.07,0.36 | -1.30 | -2.97,0.37 | -1.23 | -2.89,0.44 |
| Lag 1h | -0.64 | -2.11,0.84 | -0.82 | -2.99,1.36 | -0.88 | -2.77,1.01 | -0.84 | -2.53,0.85 | -0.71 | -2.39,0.97 |
| Lag 2h | -1.17 | -2.68,0.33 | -1.97# | -4.22,0.28 | -1.52 | -3.35,0.30 | -1.46# | -3.19,0.27 | -1.30 | -3.01,0.42 |
| Lag 3h | -1.53* | -3.05,-0.01 | -1.70 | -3.94,0.54 | -1.98* | -3.96,-0.00 | -1.90* | -3.66,-0.14 | -1.74# | -3.49,0.00 |
| Lag 4h | -1.35# | -2.87,0.17 | -1.28 | -3.54,0.99 | -1.92# | -4.13,0.29 | -1.84* | -3.61,-0.07 | -1.75# | -3.51,0.00 |
| Lag 5h | -1.47# | -3.01,0.07 | -1.33 | -3.65,1.00 | -2.51* | -4.76,-0.25 | -2.40** | -4.20,-0.60 | -2.13* | -3.91,-0.35 |
| Lag 6h | -1.27 | -2.80,0.27 | -1.01 | -3.28,1.25 | -2.27* | -4.45,-0.09 | -2.17* | -3.96,-0.38 | -2.00* | -3.77,-0.23 |
| **Black Carbon** |  |  |  |  |  |  |  |  |  |  |
| Concurrent | 2.19* | 0.09,4.29 | -0.15 | -3.05, 2.75 | -0.20 | -2.10,1.69 | -0.49 | -2.94,1.96 | -0.21 | -2.64,2.21 |
| Lag 1h | 2.18* | 0.40,3.96 | -0.38 | -2.87, 2.10 | 0.10 | -1.54,1.75 | -0.07 | -2.18,2.03 | 0.10 | -1.97,2.17 |
| Lag 2h | 0.94 | -0.67,2.55 | -2.02# | -4.38, 0.34 | -0.66 | -2.37,1.05 | -0.67 | -2.58,1.24 | -0.66 | -2.55,1.22 |
| Lag 3h | -0.23 | -1.77,1.30 | -1.80 | -4.04, 0.44 | -0.42 | -2.02,1.18 | -0.64 | -2.45,1.18 | -0.42 | -2.22,1.38 |
| Lag 4h | -1.53* | -3.04,-0.02 | -3.15** | -5.43, -0.88 | -1.03 | -2.42,0.36 | -1.36 | -3.16,0.43 | -1.03 | -2.81,0.74 |
| Lag 5h | -2.12** | -3.71,-0.53 | -3.45** | -5.82, -1.08 | -0.95 | -2.45,0.54 | -1.27 | -3.11,0.58 | -0.96 | -2.81,0.89 |
| Lag 6h | -2.04* | -3.81,-0.27 | -2.23 | -4.89, 0.43 | -1.27 | -3.10,0.57 | -1.25 | -3.30,0.80 | -1.27 | -3.30,0.76 |

SA Model #1: Additional variance term added to estimate the variance across multiple measures within the same study subject

SA Model #2: First hour of clinic visit only

SA Model #3: Generalized estimating equations used

SA Model #4: Main analysis, but relative humidity modeled with just a linear term
